# Assessment of Iron Metabolism and Inflammation in Children with Cerebral Palsy

**DOI:** 10.3390/jcm14010061

**Published:** 2024-12-26

**Authors:** Ozhan Orhan, Gul Sahika Gokdemir

**Affiliations:** 1 Department of Pediatrics, Faculty of Medicine, Mardin Artuklu University, Mardin 47100, Turkey; 2 Department of Physiology, Faculty of Medicine, Mardin Artuklu University, Mardin 47100, Turkey; gulsahikagokdemir@artuklu.edu.tr

**Keywords:** cerebral palsy, C-reactive protein, iron, systemic immuno-inflammatory index

## Abstract

**Background/Objectives:** Cerebral palsy (CP) is a motor disorder resulting from brain damage that is common in childhood. Iron is vital for the body’s basic functions. Iron metabolism disorders and inflammation contribute to the neurological complications seen in CP. The purpose of this research was to ascertain the association and correlation between markers of inflammation and iron metabolism in children with CP. **Methods**: A total of 181 children diagnosed with CP and 111 typically developing children were retrospectively included in the study. Demographic data, blood parameters, C-reactive protein, iron, total iron binding capacity, and inflammation markers were evaluated. **Results**: C-reactive protein (CRP), neutrophil-to-lymphocyte ratio (NLR) and systemic immuno-inflammatory index (SII) levels of CP children were found to be statistically significantly higher than those of control group children (*p* < 0.05). Iron (Fe) and ferritin levels were lower in the CP group, while total iron binding capacity (TIBC) was higher. Spearman correlation analysis showed significant correlations between iron, ferritin and TIBC and SII. **Conclusions:** Iron deficiency and chronic inflammation are associated with the pathophysiology of CP in patients with CP, and therefore it is important to monitor markers of iron metabolism and inflammation in these patients.

## 1. Introduction

Cerebral palsy (CP) is the most common developmental movement disorder of childhood. The disorder occurs as a result of brain injury occurring in the fetal or infantile period and usually causes non-progressive and permanent loss of motor function. CP is not limited to motor problems; it also brings sensory, cognitive, speech and behavioral problems [1].

Iron is involved in a variety of bodily cellular functions. It is required in basic functions such as deoxyribose nucleic acid (DNA) synthesis, cellular metabolism and respiration. It is also an essential component of hemoglobin and is required for oxygen transport. Iron has complex interactions with immune functions associated with infections, and iron deficiency may increase susceptibility to infections, while iron supplementation has been shown to increase the risk of some infections. Iron is a critical nutrient for almost all forms of life, and bacterial pathogens must also obtain iron from host organisms. Therefore, strict regulation of iron balance in the body is necessary [2]. Infections and chronic inflammatory conditions, which are common in children with CP, may increase the frequency of these mechanisms. It has been established that iron deficiency can impact neurodevelopment even in the absence of anemia. Iron deficiency can also affect the function of the developing brain and other organs that have high iron requirements [3,4].

Although the relationship between iron metabolism and the pathophysiology of CP is not fully understood, available data suggest that iron plays a critical role in neurological function. The brain is an organ with high energy metabolism, and iron plays an important role in the processes of oxidative phosphorylation and energy production that are essential for neurons and glial cells. Iron is also involved in the synthesis of neurotransmitters, which regulate nerve transmission. Iron deficiency can cause disturbances in these neurological processes, affecting the function of nerve cells and leading to neurodevelopmental disorders. In particular, in neurological disorders such as cerebral palsy, impaired iron metabolism can trigger oxidative stress and inflammatory processes and cause damage to brain tissue. Therefore, iron deficiency in children with CP can be considered a risk factor that may contribute to the progression of brain damage [4].

The impact of iron deficiency on the immune system and inflammatory responses is also significant and may increase the risk of infection and further complicate inflammatory processes in children with CP. Given the role of inflammation in the pathogenesis of brain damage, impaired iron metabolism may contribute to the pathophysiology of CP. Nevertheless, it is unclear if inflammation is persistent in CP or if it is only pathogenic and mainly contributes to the acute phase of injury. Although studies suggest that inflammation can persist for years in CP, leading to functional impairment throughout development and into adulthood, inflammation is a complex process and there are no clear conclusions. Research indicates that children with neurological problems often have low ferritin levels [5].

**Figure 1:**
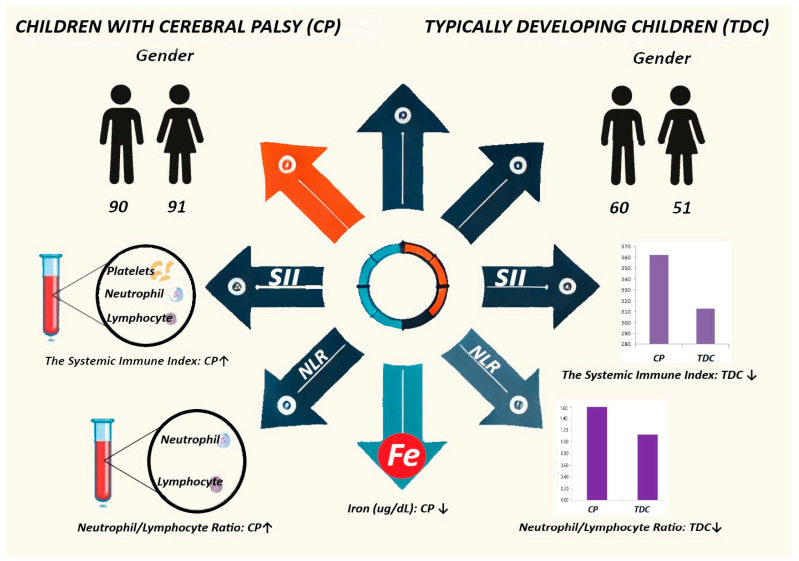
Graphical abstract showing the relationship between iron metabolism and inflammation in children with cerebral palsy (CP).

Inflammation has been shown to contribute to the pathogenesis of brain damage in CP [6]. Therefore, inflammatory processes in CP can be studied using various markers that assess inflammation. One of the markers used to assess inflammation is C-reactive protein (CRP), which is commonly used in acute and chronic inflammatory conditions. The distribution and numbers of inflammatory and pro-inflammatory cells, such as neutrophils, lymphocytes and platelets (PLT), in the peripheral blood vary depending on the release of inflammatory mediators. Parameters, such as neutrophil-to-lymphocyte ratio (NLR) and systemic immune inflammation index (SII), are inflammatory markers that can be calculated from routine hemogram values (Figure 1). Many recent studies have shown that inflammatory markers such as NLR and SII have both prognostic and diagnostic value in some diseases [7].

To our knowledge, this is the first study to investigate the relationship between iron metabolism and inflammation in children with CP. In this study, we aimed to investigate and evaluate the relationship between inflammatory markers and iron metabolism in children with cerebral palsy.

## 2. Materials and Methods

**Figure 2:**
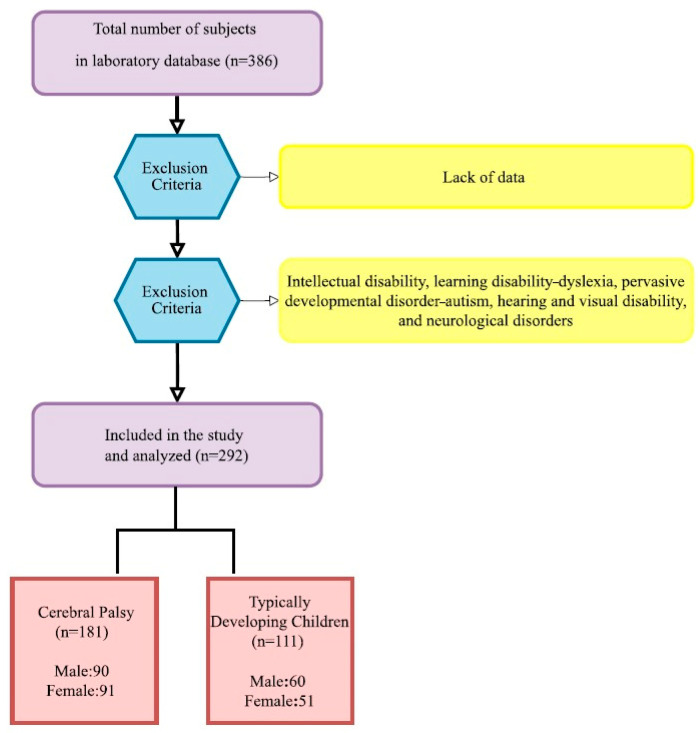
Study flowchart.

This study was designed as a retrospective study. The study included 181 patients diagnosed with CP between the ages of 5 and 18 years who applied to the Mardin Training and Research Hospital Child Health and Diseases polyclinic between 1 January 2021, and 31 July 2023, and 111 typically developing children with similar demographic characteristics who were examined as healthy children during the same period and who formed the control group. The control group was selected from routine control patients who applied to the polyclinic and did not have any chronic or surgical diseases. Children diagnosed with CP based on the International Definition and Classification of Cerebral Palsy were included in the study [8]. Hospital registration data of children with intellectual disability, learning disability—dyslexia, pervasive developmental disorder—autism, hearing and visual disability and neurological disorders other than CP were excluded from the study (Figure 2).

Demographic data (age, gender), CRP, iron (Fe), ferritin, total iron binding capacity (TIBC), white blood cells (WBC), red blood cells (RBC), hemoglobin (Hb), hematocrit (Hct), PLT, mean corpuscular hemoglobin concentration (MCHC), laboratory results data, including mean corpuscular volume (MCV), mean corpuscular hemoglobin (MCH), monocyte (MO), lymphocyte (LYMPH), and neutrophil (NEU), were recorded from patient records. NLR, neutrophil–monocyte ratio (NMR), lymphocyte–monocyte ratio (LMR) and SII were calculated mathematically.

### Statistical Analysis

The research data were analyzed in SPSS 26 software(SPSS Inc., Chicago, IL, USA) for Windows 26.0. In case of the normal distribution of the parameters, the independent sample *t* test was applied and chi-square test was applied for the evaluation of categorical variables. The Spearman correlation test was used to analyze the relationship between the data. Univariate and multivariate logistic regression analyses were performed to evaluate parameters such as age, gender, iron, ferritin, and SII between two groups of children with CP and typically developing children. Variables with *p* < 0.05 in the univariate model were included in the multivariate model as covariates. All values were presented as odds ratio (OR) and 95% confidence interval (CI) in the univariate and multivariate logistic regression models. *p* values <0.05 were considered statistically significant.

## 3. Results

The mean age of the CP group was 9.34 ± 4.16 years, while the mean age of the children with normal development group was 9.68 ± 3.32 years. No significant difference was found between the two groups in terms of age (*p* = 0.475). In the CP group, 90 were male (49.7%) and 91 were female (50.3%). In the group of children with normal development, 60 were male (54.1%) and 51 were female (45.9%). Demographically, the CP group and the typically developing children group had similar characteristics.

The median CRP level in the CP group was 1.4 (1.0–10.6), while the mean CRP level in the typically developing children group was 0.80 (0.10–2.90). There was a significant difference between the two groups in terms of CRP level (*p* < 0.001). Similarly, the median Fe level in the CP group was 52 (4–194), while it was 76 (14–158) in the typically developing group. A significant difference was found between the two groups in terms of Fe level (*p* < 0.001). In parallel with the difference in Fe levels, the TIBC level was found to be significantly lower in typically developing children than in the CP group (*p* < 0.001). The mean TIBC level in the CP group was 296.02 ± 82.10, while it was 257.35 ± 56.49 in the typically developing group. The median ferritin level was 21.60 (4.20–88.00) in the CP group and 48.80 (20.90–265.00) in the typically developing children group. Ferritin level was found to be significantly higher in typically developing children than in the CP group (*p* < 0.001). Inflammation parameters NLR and SII were significantly higher in the CP group. (*p* = 0.003, *p* = 0.012).

Significant differences were also found in the parameters of WBC, MCHC, MO, LYMPH, PLT, and NEU between the two groups, as shown in Table 1. However, RBC, HB, HCT, MCV, MCH, NMR and LMR parameters were not statistically significant (*p* > 0.05).

Table 2 shows the correlation between inflammation parameters and iron metabolism markers in children with CP and typically developing children. According to the results of Spearman correlation analysis, significant negative correlations were found between iron levels and SII (r = −0.252, *p* = 0.001) and NLR (r = −0.167, *p* = 0.024). However, no significant correlation was found between iron levels and CRP (r = 0.019, *p* = 0.799). There was no significant correlation between TIBC levels and SII (r = 0.127, *p* = 0.089) and NLR (r = −0.010, *p* = 0.897), but there was a non-significant positive correlation with CRP (r = 0.095, *p* = 0.203). No significant correlation was found between ferritin levels and SII (r = −0.130, *p* = 0.081), NLR (r = −0.069, *p* = 0.357) and CRP (r = −0.018, *p* = 0.808). In the group of children with typical development, no significant correlation was found between iron levels and SII (r = −0.083, *p* = 0.387) and NLR (r = −0.073, *p* = 0.447), but significant positive correlations were found between TDBK and SII (r = 0.167, *p* = 0.080) and NLR (r = 0.239, *p* = 0.011). No significant correlation was found between ferritin levels and SII (r = −0.065, *p* = 0.496), NLR (r = −0.027, *p* = 0.782) and CRP (r = −0.065, *p* = 0.498). These findings suggest that there are significant relationships between iron metabolism and inflammation parameters in children with CP and especially SII and NLR are significantly correlated with iron levels, whereas these correlations are weaker in typically developing children.

The median value of iron in the group with cerebral palsy was 52 (4–194). According to this value, patients with CP were divided into two groups. Inflammation values were significantly higher in the group with low iron levels (Table 3).

We used univariate and multivariate logistic regression analysis models for children with CP. Results of our multivariate model analysis are as follows: iron (OR: 0.967, 95% CI: 0.957–0.977, *p* < 0.00); ferritin (OR: 0.934, 95% CI: 0.919–0.950, *p* < 0.00); and SII (OR: 1.002, 95% CI: 1.002, *p* < 0.00) (Table 4).

## 4. Discussion

In this study, we examined various parameters to understand the relationship between iron metabolism and inflammation in children with cerebral palsy.

The biological importance of iron has been known since ancient times. Iron, which is necessary for all mammalian cells, is necessary for DNA, ribonucleic acid and protein synthesis, electron transport, cellular respiration and the structure and functions of many enzymes [6]. Iron metabolism has a significant impact on both the immune system and erythropoiesis, and inflammation can interfere with this process. Factors such as the neurological problems associated with motor problems in CP, frequent infections and malnutrition can increase inflammation and consequently iron imbalances. In order to maintain iron homeostasis, which is very important for oxygen transport, cell growth, proliferation and differentiation in the body, mechanisms related to uptake, excretion and storage are tightly controlled. However, the limited physiological excretion of iron in humans makes it more important to maintain iron homeostasis by regulating the uptake and storage functions. These physiological processes play an important role in the regulation of both cellular and systemic inflammation. Iron is ingested in the diet or as forms released from the destruction of erythrocytes. Iron transported into the cell by transferrin is taken up by endocytosis via cellular receptors and stored in the unstable iron pool in the cytosol. Excess iron is stored by ferritin or removed from the cell by ferroportin. Cellular iron homeostasis is controlled by regulatory proteins such as ferritin, hepsidin and ferroportin [9]. The hormone hepcidin controls iron absorption from the small intestine and iron release from macrophages, thus maintaining systemic iron balance. Ferritin, which stores iron in the body, rapidly binds iron and provides the iron needed for metabolism. Iron binds to transferrin in plasma and is transported to erythrocyte precursors and other cells [10].

Ferritin, which stores iron in the body, provides the iron required for metabolism by rapidly binding iron. Iron plays an important role in the regulation of oxidative stress and energy metabolism; therefore, iron deficiency can have serious effects on brain development and functions [11,12]. Iron deficiency can slow down myelination processes, especially in the developing brain, and negatively affect neurotransmitter synthesis and synaptic plasticity [12]. These processes are of critical importance in neurological disorders such as CP [1,2]. In recent years, evidence of interactions between iron deficiency and inflammation has been increasing [13]. Some studies suggest that high CRP levels in children with iron deficiency anemia may be an indicator of inflammatory processes [14]. These findings may provide an important contribution to understanding the role of iron metabolism and inflammation in CP. Therefore, evaluating iron deficiency and inflammation together in children with CP may help to better understand the pathophysiology of this disease.

Children with CP are at risk for nutritional deficiencies that are often unrelated to the degree of motor involvement [15]. Factors such as chewing, swallowing, and feeding difficulties, as well as short mealtimes, contribute to these problems [16]. Conditions such as muscle spasms and spasticity may further impede swallowing [17]. In cases of particularly severe motor involvement, malnutrition may lead to iron deficiency anemia due to inadequate iron intake and absorption. Studies have shown that children with CP have high rates of nutritional deficiencies and anemia, including iron, folate, and vitamin B12 deficiencies. Anemia in children with CP has been associated with decreased functional abilities and strength. Studies have shown that malnutrition is common in children with CP [18]. For example, a study by Almuneef et al. found that 56.4% of 74 children with CP were malnourished [19]. Ispiroğlu’s study, on the other hand, showed that iron deficiency anemia and other nutritional deficiencies are common in neurological diseases [20]. While El Shemy stated that the functional abilities of children with CP who have anemia are reduced, Chau Duc’s study revealed that anemia is mostly mild in children with CP and is seen in 60% of them [21,22].

Serum transferrin concentration increases with iron deficiency. Plasma iron concentrations are subject to significant diurnal variation and the effects of food intake. TIBC, an indicator of transferrin, is not subject to concentration changes as rapid as plasma iron concentration; therefore TIBC is inherently more stable as an indicator of iron status. Daily variations in TIBC and analysis variations are low. High TIBC levels reflect severe iron depletion. This is because high TIBC means that iron stores are low. This is associated with iron deficiency [23]. Studies of CP cases have consistently observed significantly lower levels of iron and trace elements compared with healthy controls. These reduced levels exhibit a noticeable trend, particularly in a population more susceptible to cerebral palsy. Additionally, iron deficiency anemia has been identified in individuals with CP [24,25]. In another study investigating iron deficiency anemia in CP patients, iron deficiency was detected in 40% of cases [26]. Similarly, Papadopoulos et al., in their study involving 108 CP cases, reported iron deficiency in 38% of the cases [18]. Hals et al. reported low Hb and ferritin levels in children with neuromotor impairment [27]. Ceylan reported low levels of serum iron, ferritin, B12 vitamin, and folate only in CP patients who were solely fed through oral intake. In this study, Ceylan identified significant differences among groups in complete blood count parameters (Hb, Hct, MCV, platelet) levels. However, no differences were observed in folate and ferritin levels [28]. When examined in terms of iron metabolism in our study, it was observed that the serum iron levels of children with CP were lower than those of typically developing children. At the same time, TIBC levels were found to be high in children with CP, indicating the presence of anemia. In addition, ferritin values were found to be significantly lower in our study. Although the differences in Hb and Hct values were found to be not statistically significant in our study, monocytes, platelets, lymphocytes, and neutrophils were found to be higher in children with CP than in children with normal development.

The relationship between chronic inflammation and iron metabolism in children with CP can be better understood by analyzing inflammatory markers in detail. Biomarkers such as SII, and NLR are new-generation biomarkers used to reflect the severity of inflammatory conditions and the level of systemic inflammation in the body. The fact that these inflammatory parameters can be easily calculated from routine blood tests is an advantage in that it is a simple and inexpensive method [29]. A sensitive indicator of inflammation in the human body is CRP. In addition to its inflammatory effect, CRP is considered a prognostic marker in cerebrovascular and cardiovascular diseases [30]. It is found in very small amounts in the serum of healthy individuals and does not change during the day, occasionally increasing levels are usually due to infections or trauma [31]. Demirel et al., in their study comparing CRP levels in CP with the control group, showed a statistically significant increase in CRP [32]. In Pingel et al.’s study, CRP levels were considerably higher in children with CP when compared with normative values of adults with CP, healthy adults, and typically developed children. These results demonstrate that children with cerebral palsy have higher levels of systemic inflammation [33]. Similarly, in our study, it was observed that CRP levels were significantly higher in children with CP.

Neutrophils are an important parameter of the inflammatory response and high neutrophil counts reflect inflammation [34]. Neutrophils are regulated by epithelial cells, mast cells and macrophages. Lymphocytes are important in both infection and inflammation. Platelets are involved in coagulation, hemostasis, angiogenesis and inflammatory response. SII is an index based on neutrophil, platelet and lymphocyte counts [35]. SII is a marker reflecting the balance between inflammation and immunity. It is calculated from the absolute numbers of neutrophils, lymphocytes and platelets. High SII levels indicate a strong inflammatory response in the body and are associated with various diseases such as cardiovascular disease and infections [7]. Our results show that children with cerebral palsy have higher levels of inflammation and that there may be a possible relationship between SII and CP. The correlation analysis results of our study show that there are significant relationships between iron metabolism and inflammation parameters in children with CP. In particular, inflammation markers such as SII and NLR were significantly correlated with iron levels, suggesting that inflammation may have a significant effect on iron metabolism. However, these correlations were found to be weaker in typically developing children, suggesting that there may be a relationship between iron metabolism and inflammation.

NLR is a parameter that can be easily calculated from routine blood tests. An increase in NLR is an indicator of systemic inflammation and physiological stress [36]. In a study conducted in patients with CP, NLR levels were found to be significantly higher than in the healthy group [37]. Similarly, in our study, NLR levels were significantly higher in children with CP. These findings suggest the presence of inflammatory processes and that systemic inflammation is more prominent in children with CP.

Iron plays an important role in many physiological processes and all aspects of iron homeostasis are tightly controlled. Central to this process is the regulation of iron transport. Hepsidin and its receptor, ferroportin, control the major pathways of iron transport and availability in the body. Hepsidin levels are feedback regulated by plasma iron concentration and the amount of iron stores. In addition, iron levels are negatively regulated by the activity of erythrocyte precursors, the major consumers of iron [38,39]. The regulation of hepsidin and ferroportin is affected by inflammation. In inflammatory states, pro-inflammatory cytokines increase the production and release of hepsidin. This leads to increased internalization and degradation of ferroportin and subsequent accumulation of cellular iron. This reduces circulating iron levels, which may result in insufficient iron being available to meet the body’s needs [40]. In our study, in the comparison we made among children with CP according to iron levels, the high inflammation values in children with low iron levels suggest that iron deficiency is associated with inflammation. These mechanisms may lead to functional iron deficiency and anemia by disrupting iron metabolism due to the long-term effects of inflammation in CP. The observation of low serum iron levels and high inflammatory markers (SII, NLR) in our study suggests that iron metabolism is significantly affected by systemic inflammation in children with CP.

### Study Limitations

This study has some limitations due to its retrospective design. Firstly, it lacks data on lifestyle factors such as dietary habits and physical activity levels. Secondly, the lack of data on the neurological status of the patients is another factor that limits the scope of the study. Finally, because direct causality is not always possible in retrospective studies, prospective studies are needed to further investigate the association and potential causality between inflammation and anemia. Despite these limitations, our study makes a valuable contribution to the existing literature and provides guidance for further research.

## 5. Conclusions

Our study reveals an important relationship between inflammation and iron metabolism in children with CP. Compared with typically developing children, iron levels were found to be low, TIBC was high, and inflammation markers, especially CRP, NLR, and SII, were found to be high in children with CP. As chronic inflammation and iron deficiency are thought to contribute to the pathophysiology of CP, the monitoring of inflammatory markers together with iron metabolism parameters is extremely important in the management of patients with CP. In future, more comprehensive studies, the question of whether the cognitive and motor problems of children with CP will be reduced with treatments that regulate iron metabolism and reduce inflammation should be investigated.

## Figures and Tables

**Table 1:** Comparison of demographic data and blood values in children with cerebral palsy and typically developing children.

Parameters	CP (*n* = 181)	Typically Developing Children (*n* = 111)	*p* Value
Gender (men), *n* (%)	90 (49.7%)	60 (54.1%)	0.547
Age (year), mean ± SD	9.34 ± 4.16	9.68 ± 3.32	0.475
C-reactive protein(mg/L), median (min–max)	1.4 (1.0–10.6)	0.80 (0.10–2.90)	<0.001
Iron (µg/dL), median (min–max)	52 (4–194)	76 (14–158)	<0.001
TIBC (µg/dL), mean ± SD	296.02 ± 82.10	257.35 ± 56.49	<0.001
WBC (10 × 3/µL), mean ± SD	9.00 ± 3.44	7.64 ± 2.51	<0.001
RBC (10 × 6/µL), mean ± SD	4.99 ± 0.58	5.04 ± 0.44	0.385
Hemoglobin (g/dL), mean ± SD	13.73 ± 9.26	13.36 ± 1.32	0.679
Hematocrit (%),mean ± SD	38.76 ± 4.59	39.31 ± 3.26	0.242
Platelet (10 × 3/µL), mean ± SD	315.69 ± 80.81	295.54 ± 70.95	0.031
MCHC (g/dL), mean ± SD	33.42 ± 1.63	33.95 ± 1.45	0.005
MCV (fL), mean ± SD	78.80 ± 7.10	78.14 ± 6.85	0.431
MCH (pg), mean ± SD	26.40 ± 3.09	26.40 ± 3.19	0.990
Monocyte (%), median (min–max)	0.63 (0.20–6.20)	0.52 (0.22–1.96)	<0.001
Lymphocyte (10 × 3/µL), mean ± SD	3.44 ± 1.23	3.06 ± 1.03	0.007
Neutrophil (10 × 3/µL), median (min–max)	3.46 (0.93–19.6)	3.22 (0.96–8.97)	<0.001
Ferritin (ng/mL), median (min–max)	21.60 (4.20–88.00)	48.80 (20.90–265.00)	<0.001
NMR, mean ± SD	6.64 ± 3.70	6.03 ± 2.07	0.115
LMR, mean ± SD	5.46 ± 2.34	5.89± 2.06	0.110
SII, median (min–max)	362.09 (52.65–2857.43)	312.51 (87.03–667.54)	0.012
NLR, median (min–max)	1.47 (0.21–8.55)	1.12 (0.28–2.98)	0.003

Abbreviations: CP: cerebral palsy, TIBC: total iron binding capacity, WBC: white blood cell, RBC: red blood cell, MCHC: mean corpuscular hemoglobin concentration, MCV: mean corpuscular volume, MCH: mean corpuscular hemoglobin, NMR: neutrophil/monocyte ratio, LMR: lymphocyte/monocyte ratio, SII: systemic immune–inflammation index, NLR: neutrophil/lymphocyte ratio.

**Table 2:** Correlation between inflammation parameters and iron metabolism markers in children with CP and typically developing children.

Variables	SII		NLR		CRP	
	*r*	*p*	*r*	*p*	*r*	*p*
**CP**						
**Iron**	−0.252	0.001	−0.167	0.024	0.019	0.799
**TIBC**	0.127	0.089	−0.010	0.897	0.095	0.203
**Ferritin**	−0.130	0.081	−0.069	0.357	−0.018	0.808
**Typically Developing Children**						
**Iron**	−0.083	0.387	−0.073	0.447	−0.148	0.122
**TIBC**	0.167	0.080	0.239	0.011	0.115	0.227
**Ferritin**	−0.065	0.496	−0.027	0.782	−0.065	0.498

Abbreviations: CP: cerebral palsy, CRP: C-reactive protein, TIBC: total iron binding capacity, SII: systemic immune–inflammation index, NLR: neutrophil/lymphocyte ratio.

**Table 3:** Evaluation of inflammation parameters according to iron levels in the cerebral palsy group.

Variable	Iron < 52, *n* = 82	Iron ≥ 52, *n* = 99	*p*
SII, median (min–max)	501.19 (52.64–1815.68)	318.57 (114.91–2857.42)	<0.001
NLR, median (min–max)	1.29 (0.21–5.29)	1.04 (0.37–8.55)	0.002

Abbreviations: SII: systemic immune-inflammation index, NLR: neutrophil/lymphocyte ratio.

**Table 4:** Univariate and multivariate logistic regression analysis in children with cerebral palsy.

Parameters	Univariate Model		Multivariate Model	
	OR (Cl 95%)	*p* Value	OR (Cl 95%)	*p* Value
**Age**	0.978 (0.920–1.040)	0.473		
**Gender**	1.251 (0.779–2.011)	0.354		
**Iron**	0.967 (0.957–0.977)	<0.001	0.973 (0.962–0.984)	<0.001
**Ferritin**	0.934 (0.919–0.950)	<0.001	0.929 (0.911–0.948)	<0.001
**SII**	1.002 (1.001–1.003)	0.001	1.002 (1.001–1.003)	<0.001

Abbreviations: SII: systemic immune–inflammation index, OR: Odds Ratio, CI: Confidence Interval.

## Data Availability

The datasets used and/or analyzed during the current study are available from the corresponding author on reasonable request.
